# *Neospora caninum* Evades Immunity *via* Inducing Host Cell Mitophagy to Inhibit Production of Proinflammatory Cytokines in a ROS-Dependent Manner

**DOI:** 10.3389/fimmu.2022.827004

**Published:** 2022-03-09

**Authors:** Xu Zhang, Yuru Wang, Pengtao Gong, Xiaocen Wang, Nan Zhang, Mengge Chen, Ran Wei, Xichen Zhang, Xin Li, Jianhua Li

**Affiliations:** Key Laboratory of Zoonosis Research, Ministry of Education, College of Veterinary Medicine, Jilin University, Changchun, China

**Keywords:** *N. caninum*, mitophagy, proinflammatory cytokines, ROS, immune escape

## Abstract

*Neospora caninum* is an intracellular protozoan that mainly infects cattle to cause abortion and significant economic losses worldwide. A better understanding of the immune evasion mechanisms of *N. caninum* could help to search for an effective approach to prevent and treat neosporosis. Mitophagy is used by some viruses to evade host immune surveillance. However, host cell mitophagy and its effect on *N. caninum* infection is unclear. In the present study, *N. caninum*-induced host cell mitophagy and its role in parasite infection were investigated *in vitro* and *in vivo*. Furthermore, the regulation of *N. caninum*-induced host cell mitophagy on the production of Reactive Oxygen Species (ROS), the secretions of proinflammatory cytokines, and the signals of p38, ERK, and Nlrp3 inflammasome were explored. Our results showed that autophagosomes and co-localization of LC3 with mitochondria were observed in *N. caninum-*infected macrophages. The mtDNA/nDNA ratio and the levels of mitochondrial marker proteins (Hsp60 and Tim23) were decreased with the increase of N. caninum numbers or infection time. N. caninum could induce mitophagy in brain and peritoneal lavage fluid cells of mice. Promoting mitophagy *via* mitophagy inducers (CCCP) could shorten survival time, decrease body weight, increase parasite load, and attenuate secretion of cytokines in *N. caninum* infected mice. CCCP treatment decreased the production of cytokines and Reactive Oxygen Species (ROS), and increased parasite burden in *N. caninum*-infected macrophages. Furthermore, CCCP or NAC (ROS inhibitor) treatment could inhibit ERK signal, Nlrp3 inflammasome, and cytokine production, while promote p38 signal in *N. caninum*-infected macrophages. The opposite results were obtained when using a mitophagy inhibitor (Mdivi1). Taken together, *N. caninum*-induced mitophagy could regulate the activations of p38, ERK, Nlrp3 inflammasome to inhibit the production of inflammatory cytokines in a ROS-dependent manner to escape host immune surveillance.

**Graphical Abstract:**
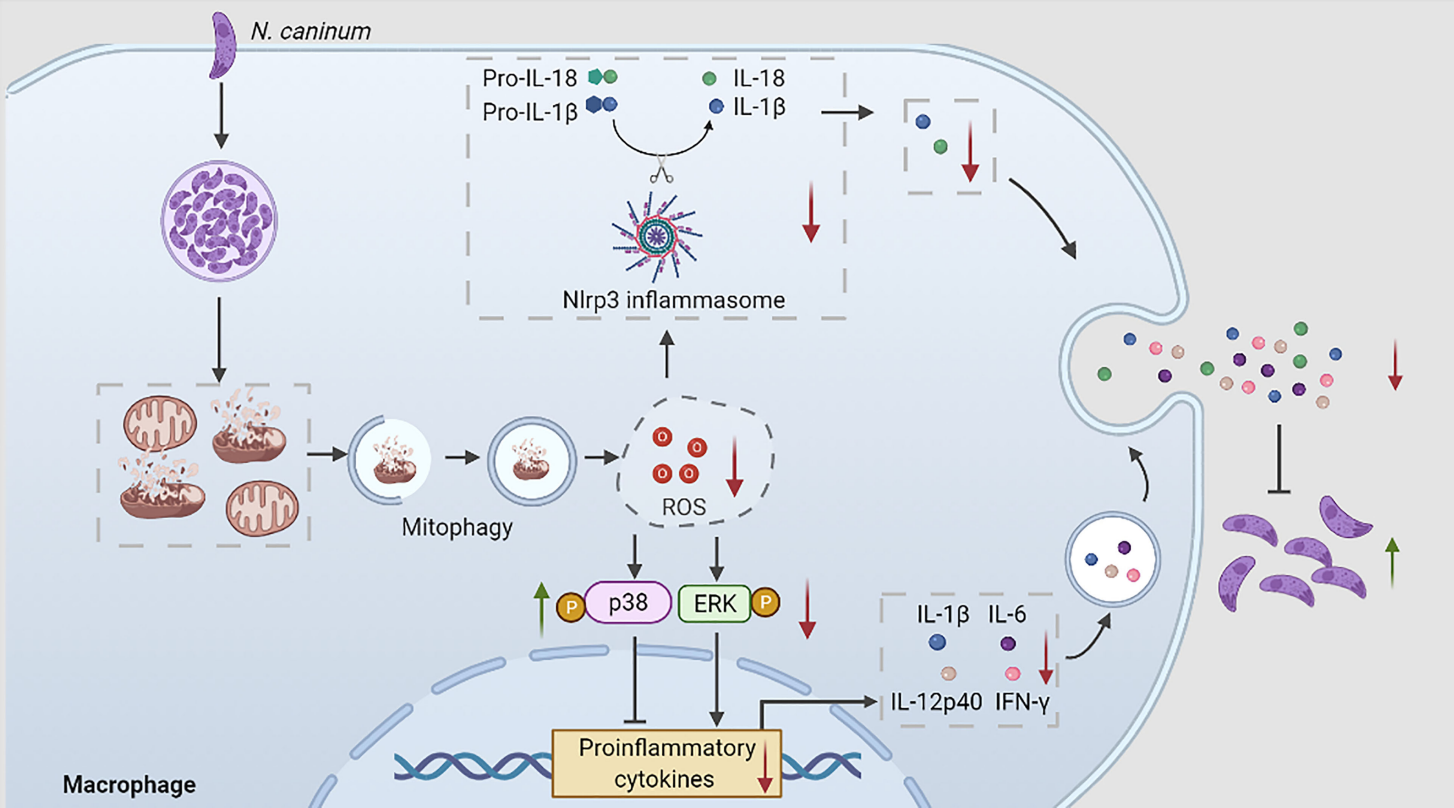
During N. caninum infection, N. caninum could induce host cell mitophagy, inhibit ROS production, which regulated p38, ERK, and Nlrp3 inflammasome signals to reduce the secretions of proinflammatory cytokines to escape host clearance.

## 1 Introduction

Neosporosis, caused by *N. caninum*, an obligate intracellular protozoan, is recognized as one of the major causes of abortion in cattle worldwide, resulting in significant economic losses in the cattle industry (1). The definitive hosts of *N. caninum* are dogs and other canids, while its intermediate hosts take a wide range of domestic and wild animals (1). *N. caninum* has been reported in more than 16 countries (2), which indicated widespread exposure and a potential public health problem. Previous studies have emphasized on immune response against *N. caninum* infection. Both innate and adaptive immunities played crucial roles in defending *N. caninum* infection (3–6). Although some potential vaccine candidates have been developed, overall, effective drugs and vaccines are still urgently needed to treat neosporosis.

Some protozoa have evolved various strategies to evade host immune system (7, 8). *Toxoplasma gondii* could control host signaling pathways such as STAT1, NF-κB signals, and caspase-1 cleavage to decrease the production of cytokines such as IFN-γ and IL-1β (9). High virulence *Trypanosoma cruzi* strains (Colombian/Tc-I and Y/TcII) could inhibit the expressions of TLR2, TLR4, TLR9, TRIF, and Myd88, leading to decreased IL-12 production in mice (10). *Leishmania* could inhibit NF-κB, ERK, JNK, and p38 signals to reduce the production of TNF-α and IFN-γ in macrophages (11, 12). *N. caninum* could promote p38 phosphorylation to inhibit the host’s innate immune responses in mouse macrophages (13). Further investigation of the immune evasion mechanisms of *N. caninum* will help us to understand the pathogenesis and design new strategies for the prevention and treatment of neosporosis.

As an essential energy generator for cell homeostasis, mitochondria are an important channel for programmed cell death (14). This core function requires the quality of mitochondria to be strictly controlled. The term of mitophagy was first coined by *John Lemasters* in 2005, which refers to the selective autophagic degradation of mitochondria to promote the clearance of damaged mitochondria (15). The damaged mitochondria contain series of damage-associated molecular patterns, such as mitochondrial DNA (mtDNA), mitochondrial ROS, and N-formylated peptides, which are released into the cytoplasm following cellular necrosis and pathogens invasion to initiate inflammatory responses (16). Mitophagy is the targeted phagocytosis and destruction of mitochondria by autophagosomes, which is generally considered to be the main regulatory mechanism of the mitochondrial quality control process and involved in regulating host immune responses (17). Moreover, mitophagy has been demonstrated to be used by certain viruses and bacteria to escape from host immune clearance (18–20). However, mitophagy occurrence has not been found in the host during parasites infection and whether protozoa could promote host mitophagy to evade host clearance has not been reported.

It is noted that the secretions of IL-1β, IL-6, IL-12p40, IFN-γ, IL-18, and TNF-α played essential roles against *N. caninum* infection (4–6). Previous studies showed MAPK signal, NF-κB signal, and NLRP3 inflammasome regulated production of proinflammatory cytokines to control *N. caninum* infection (4, 21, 22). ROS contributed to the production of proinflammatory cytokines (23) and mitophagy played an important role in ROS scavenging (16, 24). Recent evidences implicated that mitophagy eliminates dysfunctional mitochondria to restrict inflammatory cytokine secretions by inhibiting inflammasome, NF-κB signal, and so on (7). HIV ssRNA inhibited mitophagy to promote the release of proinflammatory and neurotoxic cytokines *via* Nlrp3 inflammasome in microglia (25). Mitophagy could reduce mtDNA release aggravated stretching-induced inflammation and lung epithelial cell injury *via* the TLR9/MyD88/NF-κB pathway (26). However, whether *N. caninum* infection could promote host mitophagy, or whether mitophagy plays a role in *N. caninum* survival and in the regulation of host proinflammatory response have not been extensively investigated.

In the present study, *N. caninum*-induced host cell mitophagy and its role in parasite infection were investigated *in vitro* and *in vivo*. Furthermore, the regulation mechanism of host cell mitophagy on ROS, cytokines and signal pathways were explored.

## 2 Materials and Methods

### 2.1 Mice and Peritoneal Macrophages

Wild-type (WT) female 6-8-week-old C57BL/6 mice were purchased from Liaoning Changsheng Experimental Animal Centre. Nlrp3^-/-^ mice with C57BL/6 genetic background were obtained from the Jackson Laboratory (4). WT or Nlrp3^-/-^ mice were intraperitoneally injected with 2 ml of 5% thioglycolate medium (BD Biosciences, CA, USA) per mouse, and 3 days later peritoneal macrophages (PMs) from the peritoneal cavity were collected by flushing twice with 6 ml ice sterile PBS. PMs were counted and plated into 6-well-plate at 2.5×10^6^ cells per well with RPMI-1640 medium supplemented with 10% fetal bovine serum (FBS). The supernatant was discarded the next day, and adherent cells were further cultures as PMs (27).

### 2.2 Parasites and Excretory Secretory Products (ESPs)

*N. caninum* used in the present study was Nc-1 strain and was maintained in Vero cells with RPMI-1640 medium supplemented with 2% FBS (BI, Israel) in a 5% CO_2_ atmosphere at 37°C. *N. caninum* tachyzoites were harvested by gradient density centrifugation with 40% Percoll solution (Sigma, Shanghai, China) at 1500×g for 30 min. The precipitate was collected and washed twice with RPMI-1640 medium, while the supernatant was discarded. The numbers of tachyzoites were determined using a hemocytometer. Excretory secretory products (ESPs) of *N. caninum* were prepared and stored as previously reported (28). And the concentration of ESPs was determined using the BCA Protein Assay Kit (Thermo Fisher Scientific, Waltham, MA).

### 2.3 Antibodies

Mouse anti-Tim23 was from Santa Cruz Biotechnology (CA, USA). Rabbit anti-Hsp60 antibody, FITC-conjugated anti-rabbit IgG antibody, and HRP-conjugated anti-mouse, rabbit, or goat IgG antibodies were purchased from Proteintech (Wuhan, China). Rabbit anti-β-actin, anti-LC3, anti-ERK, anti-p38, anti-p65, anti-phospho-ERK (Thr202/Tyr204), anti-phospho-p38 (Thr180/Tyr182), and anti-phospho-p65 (Ser536) antibodies were obtained from Cell Signaling Technology (Danvers, MA, USA). Goat anti-IL-1β antibody was from R&D (Minneapolis, USA). Mouse anti-caspase-1 (p20) and mouse anti-Nlrp3 antibodies were purchased from Adipogen (Liestal, Switzerland).

### 2.4 Mitophagy Detection

#### 2.4.1 Transmission Electron Microscopy (TEM)

PMs were stimulated with *N. caninum* at a multiplicity of infection (MOI) of 1:3 for 16 h and PMs were treated with Carbonyl cyanide 3-chlorophenylhydrazone (CCCP) (10 µM, Sigma-Aldrich, MO, USA) for 16 h as the positive control. The PMs were trypsinized, centrifuged, and collected. The cells were fixed in 5% (w/v) glutaraldehyde, then post-fixed in 1% (w/v) osmium tetroxide, dehydrated by the concentration gradient of ethanol (50%, 70%, 80%, 90%, and 95%), and embedded by Epon812. Then, sections were cut at 0.12 μm thickness and stained using 1% (w/v) uranyl acetate and 0.2% (w/v) lead citrate. The autophagosomes were observed by TEM (HITACHI, Japan).

#### 2.4.2 Immunofluorescence

PMs plated on glass coverslips in 24-well culture plates (5×10^5^ per well) were stimulated either with *N. caninum* at a MOI of 1:3 for 16 h or with CCCP (10 µM). Mito Tracker Red (Thermo Fisher Scientific, Waltham, MA, USA) was used to label mitochondria according to the instructions. After washing twice with PBS, cells were fixed for 15 min at room temperature with 4% formaldehyde in PBS and then permeabilized with 0.1% Triton X-100 in PBS for 10 min. After blocking with 3% BSA for 1 h at room temperature, cells were incubated with the primary antibody to LC3 (1:100 diluted 3% BSA) overnight at 4°C followed by incubation with FITC anti-rabbit secondary antibody diluted at 1:1000. Cells were eventually counter stained with DAPI for 5 min. Immunofluorescence-stained cells were observed with a Zeiss LSM 710 confocal microscope (Carl Zeiss). For the quantification of mitophagy, the number of PMs in which endogenous LC3 co-localized with Mito Tracker were evaluated per 50 cells.

#### 2.4.3 GFP-LC3 Fluorescence Analysis

Human kidney epithelial 293T cells were maintained in our laboratory. 293T cells were seeded in 24-well-plate at 5×10^5^ cells per well with DMEM supplemented with 10% FBS (BI, Israel). The cells were transfected with pEGFP-LC3 and pEGFP empty vector by using Lipofectamine 2000 transfection reagent (Invitrogen, USA). 24 h after transfection, the cells were stimulated with *N. caninum* at a MOI of 1:3 for 16 h. Mito Tracker Red was used to label mitochondria and DAPI for nuclear. Cells were observed with a Zeiss LSM 710 confocal microscope (Carl Zeiss). For the quantification of mitophagy, the number of 293T cells in which GFP-LC3 co-localized with Mito tracker were counted per 50 cells.

#### 2.4.4 Detection of mtDNA/nDNA Ratios and Mitochondrial Marker Proteins *In Vivo* and *In Vitro*

PMs were stimulated with *N. caninum* at a MOI of 1:1 for 8 h, 16 h, and 24 h, respectively. PMs were also either stimulated with different MOIs (cell:parasite = 1:1, 1:3, 1:5) of *N. caninum* for 16 h, or stimulated with different concentrations (50 µg/ml, 100 µg/ml, 200 µg/ml) of ESPs. In addition, PMs were treated with CCCP (10 µM) for 8 h as the positive control. Then DNA and protein of the PMs were extracted to measure mtDNA/nucleic DNA (nDNA) and mitochondrial marker proteins (Hsp60 and Tim23).

In mouse experiments, 15 WT C57BL/6 mice (6-8-week-old) were infected intraperitoneally with 2.5×10^7^ tachyzoites diluted in 100 µl sterile PBS per mouse and 3 mice were injected intraperitoneally with 100 µl sterile PBS as the negative control. From the third day to the seventh day post-infection (d p.i.), 3 mice/day were euthanized and the brain tissues were collected to detect Hsp60 and Tim23. Peritoneal lavage cells were collected from peritoneal lavage fluid by centrifuging for 5 min at 500 g and the brain tissues were homogenized. The DNA was extracted and mtDNA/nDNA ratios were measured in the peritoneal lavage cells and brain tissues.

To measure Hsp60 and Tim23, the PMs and ground brain tissues were lysed by RIPA buffer (Beyotime Biotechnology, Shanghai, China) and the protein concentration was determined using the BCA Protein Assay Kit. 30 μg protein samples were analyzed by SDS-PAGE and then transferred onto 0.22 μm polyvinylidene fluoride membranes (Millipore, MA, USA). Membranes were blocked in 5% skim milk, incubated overnight at 4°C with primary monoclonal antibodies of anti-Hsp60 (1:1000), anti-Tim23 (1:100), and anti-β-actin (1:1000). The membranes were incubated with anti-mouse or rabbit secondary antibodies (1:5000) for 1 h at room temperature. After washing by TBST, the membranes were visualized using the ECL Western blot Detection System (Clinx Science Instruments Co., Ltd., Shanghai, China). The Relative Gray Value (target protein/internal reference) of Western blot bands was analyzed by Image J (Image J Software, National Institutes of Health, Bethesda, MD, USA).

To quantify the mtDNA/nDNA ratios, qPCR was used to amplify mtATP6 gene (from the mitochondrial genome) and Rpl13a gene (from the nuclear genome), and the protocols were performed as previously described (20). The mtDNA/nDNA ratios was calculated by a comparative Ct method, using the following equation: mtDNA/nDNA = 2^−ΔCt^. Primer sequences were as follows: mouse mtATP6: forward: 5′-CAGTCCCCTCCCTAGGACTT-3′, reverse: 5′-TCAGAGCATTGGCCATAGAA-3′; mouse Rpl13a: forward: 5′-GGGCAGGTTCTGGTATTGGAT-3′, reverse: 5′-GGCTCGGAAATGGTAGGGG-3′.

### 2.4 Animal Infection Experiment

The mice were randomly divided into seven groups, including Mdivi1+*N. caninum* group (10 mice), CCCP+*N. caninum* group (10 mice), solvent+*N. caninum* group (10 mice), and *N. caninum* group (10 mice). The mice were infected intraperitoneally with 2.5×10^7^ *N. caninum* tachyzoites diluted in 100 µl PBS. After 24 h, Mdivi1 (MCE, Shanghai, China) and CCCP was diluted in PBS, and mice were then immediately injected intraperitoneally with Mdivi-1 (50 mg/kg body weight/per day) or with CCCP at a dose of 5 mg/kg body weight/per day (20). The mice were monitored and weighed each day. Then the mice were euthanized at 5 d p.i. and the brain, heart, liver, spleen, kidney, and lung tissues were collected to measure parasite burden. The serum was collected to detect cytokines at 5 d p.i. Mdivi1 group (3 mice as inhibitor control), CCCP group (3 mice as inhibitor control) and PBS group (3 mice as negative control).

### 2.5 Inhibitor Experiment

PMs were pretreated with Mdivi1 (20 µM), CCCP (10 µM) (20), and ROS inhibitor (NAC; 2 mM, Selleck, Shanghai, China) for 2 h (29) before being stimulated by *N. caninum* for 30 min to detect the phosphorylation of p38, ERK, and NF-κB p65. PMs were also stimulated by *N. caninum* for 24 h to measure Nlrp3, Caspase1, and IL-1β by Western blot as previously described (30). PMs were pretreated with Mdivi1 (20 µM), CCCP (10 µM), NAC (2 mM), p38 inhibitor (SB203580; 5 μM, Sigma-Aldrich, MO, USA), and ERK inhibitor (PD98059; 5 μM, Sigma-Aldrich, MO, USA) for 2 h, followed by stimulation with *N. caninum* for 24 h to detect parasite burden by qPCR and the production of cytokines by ELISA.

### 2.6 Western Blot Analysis

The cell lysates and supernatants of PMs were collected and assessed by Western blot as described previously (28). The rabbit monoclonal anti-β-actin, anti-ERK, anti-p38, anti-p65, anti-phospho-ERK (Thr202/Tyr204), anti-phospho-p38 (Thr180/Tyr182), anti-phospho-IκBα (Ser32), anti-phospho-p65 (Ser536), mouse monoclonal anti-Caspase1 and anti-NLRP3, and goat monoclonal anti-IL-1β diluted at 1:1000 with 5% BSA were used to incubate the membranes overnight at 4°C. Then the membranes were incubated with anti-rabbit/mouse/goat secondary HRP-conjugated antibodies (Proteintech, Wuhan, China) diluted at 1:5000 with 5% skim milk. Western blot bands were detected using the enhanced chemiluminescence reagent (Vigorous, Beijing, China). The membranes were visualized using the ECL Western blot Detection System.

### 2.7 *N. caninum* DNA Detection by qPCR

Parasite replication in the infected cells and mouse homogenized tissues was monitored as previously described by performing qPCR analysis of the parasite DNA (4). Genomic DNA from 1×10^7^ tachyzoites of *N. caninum* and total DNA from infected cells and mouse tissues were extracted using a Genomic DNA Extraction Kit (TIANGEN, Beijing, China) following the manufacturer’s protocol. The total DNA from infected cells (200 ng) and tissues (500 ng) was analyzed by qPCR with FastStart Universal SYBR Green Master reagent (Roche Diagnostics, Mannheim, Germany), and a 76-bp fragment of *N. caninum* DNA was amplified using the following primers: forward (5′-ACTGGAGGCACGCTGAACAC-3′); reverse (5′-AACAATGCTTCGCAAGAG GAA-3′). The number of parasites was determined based on a standard curve obtained using DNA from serial dilutions of *N. caninum* tachyzoites (from 1×10^7^ to 1×10^0^ parasite).

### 2.8 ELISA Analysis

Supernatants from cell culture and serum from mice were measured by mouse IL-1β, IL-18, IFN-γ, IL-12p40, IL-6, or TNF-α Ready-Set-Go Kits (eBioscience, San Diego, CA, USA) according to the manufacturer’s instructions.

### 2.9 ROS Detection

PMs were pretreated with Mdivi1 (20 µM), CCCP (10 µM), NAC (2 mM) for 2 h, followed by stimulation with *N. caninum* (MOI 1:3) for 3 h or with Rosup (Beyotime Biotechnology, Shanghai, China) according to the instructions as the positive control. Then the supernatant was removed, and the PMs were incubated with the DCFH-DA (Beyotime Biotechnology, Shanghai, China) for 20–30 min at 37°C, followed by 3 washes with FBS-free RPMI in the dark as the manufacturer’s instructions. Intracellular ROS were analyzed using a FACS Aria flow cytometer (BD Biosciences, CA, USA).

### 2.10 Statistical Analysis

The data were expressed as mean ± SD. Data sets with only two independent groups were analyzed for statistical significance using unpaired, two-tailed Student’s T-test. Data sets with more than two groups were analyzed using one-way ANOVA with Tukey-Kramer *post hoc* test. All graphs were generated using GraphPad Prism 5 (GraphPad Software, Inc., San Diego, CA, USA). All p values less than 0.05 were considered significant (*p<0.05, **p<0.01, ***p<0.001). P values equal to or more than 0.05 were considered no significant (p>0.05, ns). All data of this study were obtained from 3 independent experiments.

## 3 Results

### 3.1 *N. caninum* Induced Mitophagy Occurred Both *In Vitro* and *In Vivo*

**Figure 1:**
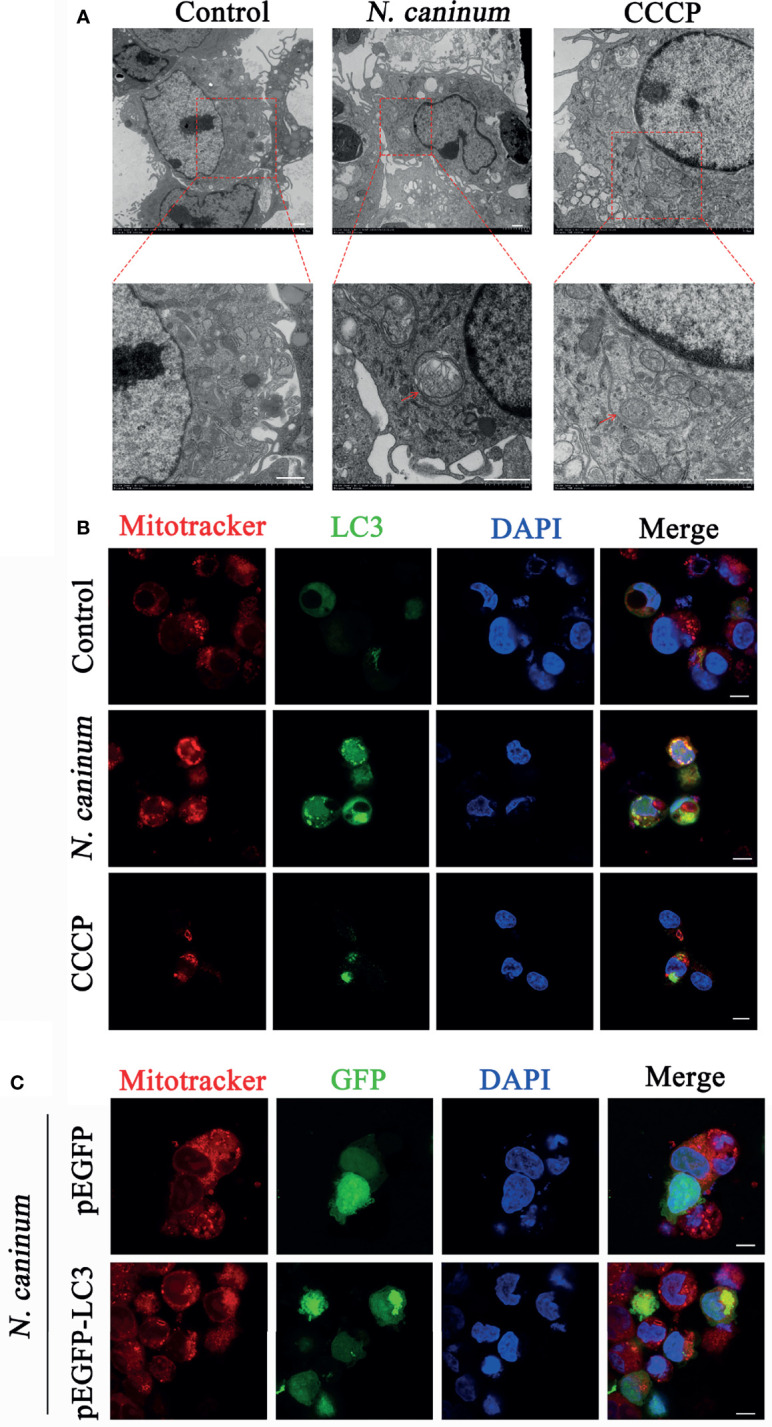
Autophagosomes and co-localization of LC3 with mitochondria were observed in *N. caninum-*stimulated PMs by TEM and IFA. **(A, B)** PMs were stimulated with *N. caninum* at MOI 1:3 for 16 h, with CCCP (10 µM) treatment as the positive control, and the medium only as the negative control. **(A)** Autophagic bodies were observed by TEM assay and red arrows pointed to autophagosomes. **(B)** The co-localization of LC3 (green) with mitochondria (red) was observed by immunofluorescence and the nucleus was blue. **(C)** 293T cells were transfected with pEGFP-LC3 vector and pEGFP empty vector for 24 h by using Lipofectamine 2000 transfection reagent. Then the cells were stimulated with *N. caninum* at MOI 1:3 for 16 h. The co-localization of GFP-LC3 (green) with mitochondria (red) was observed by immunofluorescence and the nucleus was blue (Scale bar=10 µm).

**Figure 2:**
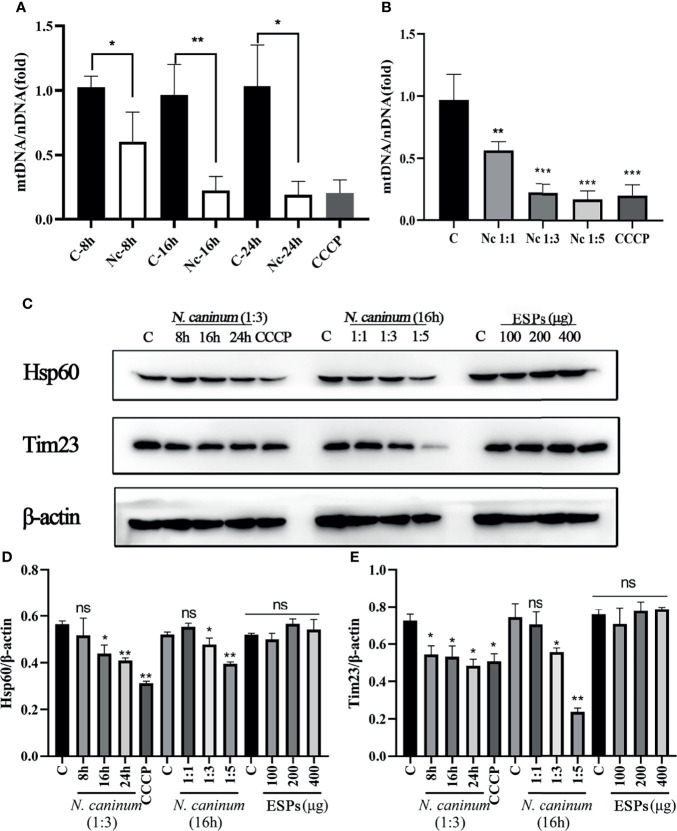
The level of *N. caninum*-induced mitophagy was dependent on MOI- and time-dependent manners. PMs were stimulated with *N. caninum* at MOI 1:1 for 8 h, 16 h, and 24 h, with different MOI (cell:parasite = 1:1, 1:3, 1:5) of *N. caninum* for 16 h, and with different concentrations of ESPs (50 µg/ml, 100 µg/ml, 200 µg/ml). PMs were treated with CCCP (10 µM) for 8 h as the positive control. The mtDNA/nDNA ratios in PMs treated with different time **(A)** and different MOI **(B)** were measured by qPCR analysis. **(C)** The expressions of mitochondrial marker proteins (Hsp60 and Tim23) were measured by Western blot and **(D, E)** relative gray of Western blot in panes was analyzed by Image J. One-way ANOVA assay with Tukey-Kramer *post hoc* test was used for analyzing mtDNA/nDNA ratios and the relative gray of Western blot in panes. Data are expressed as the mean ± SD from three independent experiments (*p<0.05, **p<0.01, ***p<0.001, ns represents no significant differences).

TEM and Immunofluorescence experiments were performed to determine whether mitophagy occurred in mouse macrophages during *N. caninum* infection. Results showed that mitochondria were enclosed by autophagosome in *N. caninum-*stimulated or CCCP treated (mitophagy inducer) PMs by TEM (**Figure 1A**). Immunofluorescence results demonstrated that N. caninum induced co-localization of mitochondria with endogenous LC3 in PMs and with GFP-LC3 in 293T cells (**Figures 1B, C**). And *N. caninum* induced mitophagy occurrence in 32.6% PMs and 31.0% 293T cells (**Figures S1A, B**). To evaluate whether *N. caninum*-induced mitophagy was dependent on infecting numbers of *N. caninum* or infection time, the levels of mtDNA and the amount of mitochondrial inner membrane protein Tim23 and matrix protein Hsp60 in mouse PMs were measured. With the increase of the numbers of *N. caninum*- or infection time, the mtDNA/nDNA ratios and the levels of Hsp60 and Tim23 protein decreased gradually, which was similar to that of CCCP treatment (**Figures 2A–C**). In addition, Western blot results were further analyzed by grayscale analysis (**Figures 2D, E**). However, *N. caninum* ESPs had no impact on the expression levels of Hsp60 and Tim23 in PMs (**Figures 2C–E**). Altogether, our data demonstrated that *N. caninum* induced mitophagy in mouse macrophages.

**Figure 3:**
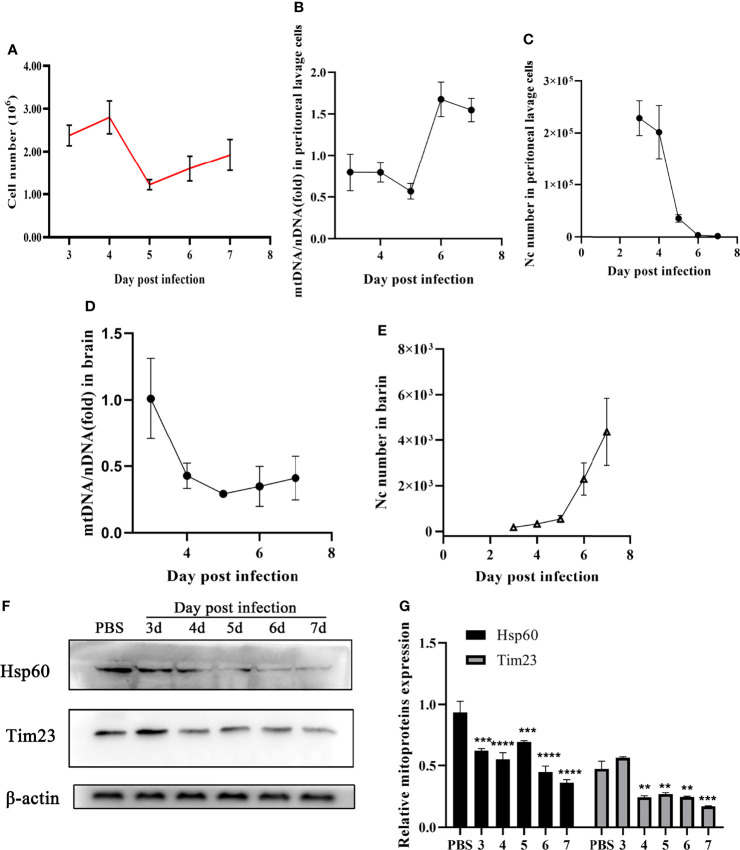
*N. caninum* induced mitophagy in the brain and peritoneal lavage cells of mice. 15 mice were injected intraperitoneally with 2.5×10^7^ tachyzoites diluted in 100 µl sterile PBS per mouse and 3 mice were injected intraperitoneally with 100 µl sterile PBS as the negative control. From the third day to the seventh d p.i., the mice (3 mice/day) were euthanized and the peritoneal lavage cells and brain were collected to detect parasite number, mtDNA/nDNA ratios, and mitochondrial marker proteins (Hsp60 and Tim23). **(A)** The number of cells in peritoneal lavage cells was measured. The mtDNA/nDNA ratios of cells from peritoneal lavage cells **(B)** and brain tissue **(D)**. The number of *N. caninum* in peritoneal lavage cells **(C)** and brain **(E)** were detected by qPCR. **(F)** The expressions of Hsp60 and Tim23 in the brain tissue were measured by Western blot and **(G)** relative gray of Western blot in panes was analyzed by Image J. One-way ANOVA assay with Tukey-Kramer *post hoc* test was used for analyzing the relative gray of Western blot in panes. Data are expressed as the mean ± SD from three independent experiments (**p<0.01, ***p<0.001, ****P<0.0001).

Furthermore, mitophagy was identified in mice during N. caninum infection. No significant difference in total cells counts was observed in peritoneal lavage fluids (**Figure 3A**). With the decrease of *N. caninum* number, the levels of mtDNA/nDNA ratios gradually increased from the third to seventh days post-infection in peritoneal lavage cells during *N. caninum* infection (**Figures 3B, C**). However, with the increase of parasite load in the brain, the levels of mtDNA and the protein expression levels of Hsp60 and Tim23 showed a downward trend (**Figures 3D–F**). Western blot results were further analyzed by grayscale analysis (**Figure 3G**). Taken together, these results indicated that mitophagy occurred in *in vitro* and *in vivo* during *N. caninum* infection.

### 3.2 *N. caninum*-Induced Host Mitophagy Promoted Parasite Loads in Mice

**Figure 4:**
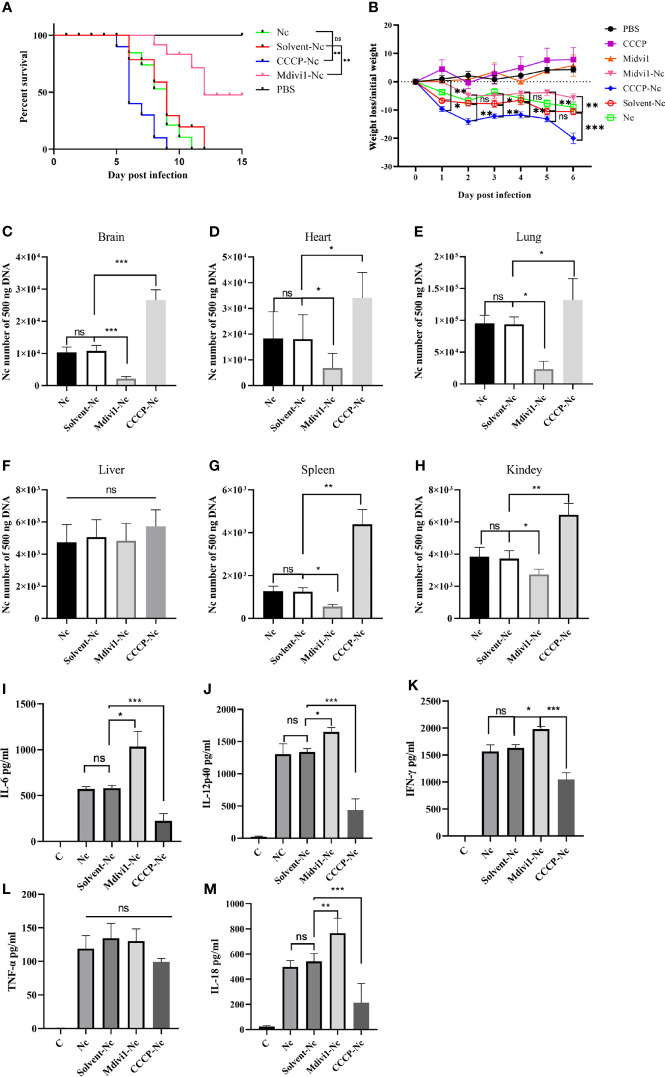
Mitophagy could shorten survival time, decrease body weight, increase parasite load, and attenuate secretions of cytokines in mice during *N. caninum* infection. Mice were injected intraperitoneally with 2.5×10^7^ *N. caninum* tachyzoites diluted in 100 µl PBS. After 24 h, the mice were then immediately injected intraperitoneally with Mdivi1 (50 mg/kg body weight) and with CCCP (5 mg/kg body weight). **(A)** The survival of mice was monitored for 15 days after *N. caninum* infection. During infection, 2 out of 10 mice per group in the CCCP-Nc, solvent-Nc, and Nc groups were euthanized due to excessive body weight loss (>20%). And Kaplan–Meyer curve-analysis was performed for survival. **(B)** The weight of mice was recorded daily for 6 days. And two-way ANOVA with Bonferroni posttests was performed to analyze the statistical significance of weight loss. On the fifth-day post infection, the numbers of *N. caninum* in the brain **(C)**, heart **(D)**, lung **(E)**, liver **(F)**, spleen **(G)**, kidney **(H)** samples were detected by qPCR. The production of IL-6 **(I)**, IL-12p40 **(J)**, IFN-γ **(K)**, TNF-α **(L)**, and IL-18 **(M)** in serum were quantified by ELISA on the fifth day post infection. One-way ANOVA assay with Tukey-Kramer *post hoc* test was used for analyzing the number of *N. caninum* and production of cytokines. Data are expressed as the mean ± SD from three independent experiments (*p<0.05, **p<0.01, ***p<0.001, ns represents no significant differences).

**Figure 5:**
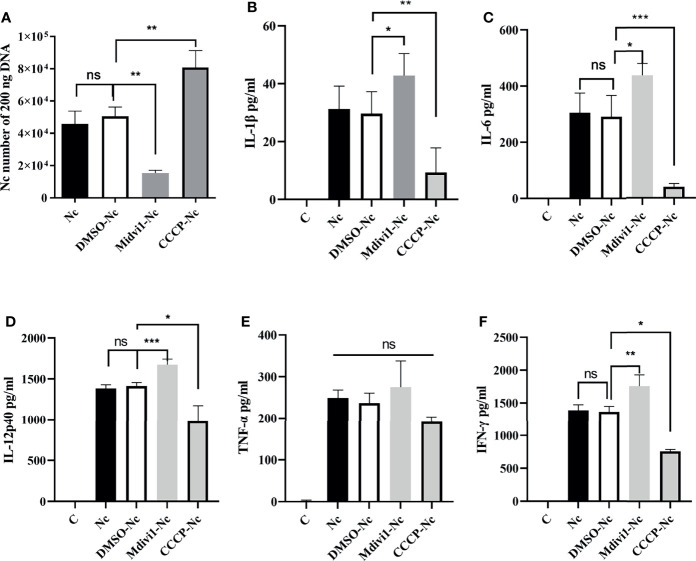
*N. caninum*-induced mitophagy inhibited the production of inflammatory cytokines in PMs. PMs were pretreated with Mdivi1 (20 µM) and CCCP (10 µM) for 2 h and were stimulated with *N. caninum* at MOI 1:3 for 24 h. **(A)** The number of parasites was evaluated by qPCR. The production of IL-1β **(B)**, IL-6 **(C)**, IL-12p40 **(D)**, TNF-α **(E)**, and IFN-γ **(F)** were detected by ELISA. One-way ANOVA assay with Tukey-Kramer *post hoc* test was used for analyzing numbers of *N. caninum* and production of cytokines. Data are expressed as the mean ± SD from three independent experiments (*p<0.05, **p<0.01, ***p<0.001, ns represents no significant differences).

To further determine the effects of mitophagy on *N. caninum* infection, mitophagy inducer (CCCP) and inhibitor (Mdivi1) were used both *in vivo* and *in vitro*. We found that Mdivi1 treatment increased the survival rate of mice while CCCP treatment accelerated the time to death of mice during *N. caninum* infection (**Figure 4A**). The CCCP-treated mice exhibited a significant loss in body weight compared with untreated mice during *N. caninum* infection. On the contrary, Mdivi1 alleviated the weight loss in mice (**Figure 4B**). Subsequently, we examined the parasite burden of different tissues by qPCR, and the parasite loads in the brain (2.46-fold), heart (1.89-fold), lung (1.41-fold), spleen (3.51-fold), and kidney (1.73-fold) in mice treated by CCCP were significantly increased compared to that in the solvent+*N. caninum* mice (**Figures 4C–E, G, H**). There was no difference in the parasite load in the liver (**Figure 4F**). Moreover, Mdivi1 treatment inhibited *N. caninum* burden in the brain (20%), heart (38%), lung (25%), spleen (44%), and kidney (74%) but not in the liver (**Figures 4C–H**). In addition, the number of *N. caninum* in mouse PMs treated with Mdivi1 (30%) was reduced, while the number was increased with CCCP treatment (1.60-fold) during *N. caninum* infection (**Figure 5A**). Taken together, these results indicated that *N. caninum*–induced mitophagy played a critical role in parasite survival in mice.

### 3.3 *N. caninum*-Induced Mitophagy Decreased the Production of IL-1β, IL-6, IL-12p40, IFN-γ, IL-18, and TNF-α *In Vivo* and *In Vitro*

It is noted that the secretions of IL-1β, IL-6, IL-12p40, IFN-γ, IL-18, and TNF-α played essential roles against *N. caninum* infection (4–6). Thus, we examined whether the role of mitophagy in parasite survival was through inhibiting cytokines production. We found that Mdivi1 treatment enhanced the production of IL-6 (1035.54 ± 165.21 pg/ml), IL-12p40 (1654.33 ± 39.62 pg/ml), IFN-γ (1981.31 ± 49.41 pg/ml), and IL-18 (765.21 ± 59.81 pg/ml), while CCCP treatment suppressed the production of IL-6 (222.67 ± 64.78 pg/ml), IL-12p40 (435.21 ± 87.52 pg/ml), IFN-γ (1046.76 ± 128.61 pg/ml), and IL-18 (213.65 ± 68.35 pg/ml) in mice during *N. caninum* infection compared to the solvent+*N. caninum* group (580.57 ± 29.60 pg/ml, 1338.57 ± 31.59 pg/ml, 1633.27 ± 62.69 pg/ml, 542.12 ± 60.68 pg/ml) (**Figures 4I, J, K, M**). Moreover, the production of IL-1β, IL-6, IL-12p40, IFN-γ were increased in Mdivi1-treated PMs (42.85 ± 4.39 pg/ml, 438.03 ± 41.80 pg/ml, 1674.33 ± 66.86 pg/ml, and 1755.52 ± 170.31 pg/ml), while were inhibited in CCCP-treated PMs (10.07 ± 2.76 pg/ml, 39.78 ± 4.78 pg/ml, 982.21 ± 90.11 pg/ml, and 763.62 ± 47.06 pg/ml) compared with solvent-treated PMs (291.35 ± 33.16 pg/ml, 1413.22 ± 43.48 pg/ml, 1365.22 ± 83.60 pg/ml) infected by *N. caninum* (**Figures 5B–D, F**). However, there were no significant differences in TNF-α production both *in vivo* and *in vitro* (**Figures 4L**, **5E**). These data suggested that N. caninum-induced mitophagy could inhibit the production of proinflammatory cytokines in PMs and mice during *N. caninum* infection.

### 3.4 *N. caninum*-Induced Mitophagy Suppressed Secretions of Proinflammatory Cytokines in a ROS-Dependent Manner

**Figure 6:**
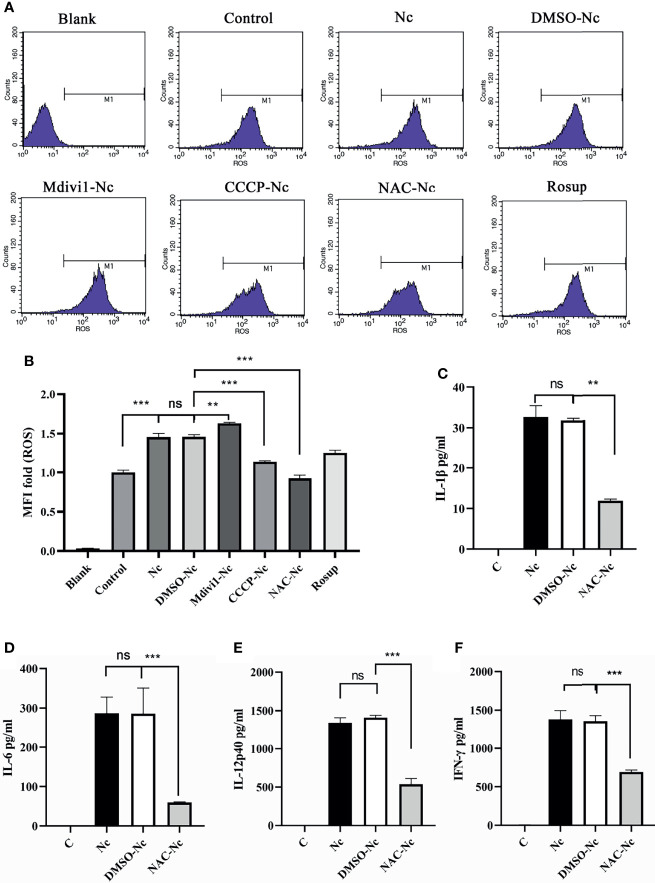
*N. caninum*-induced mitophagy inhibited the production of inflammatory cytokines in a ROS-dependent manner in PMs. PMs were pretreated with Mdivi1 (20 µM), CCCP (10 µM), and ROS inhibitor (NAC, 2 mM) for 2 h, and the PMs were treated with *N. caninum* (MOI 1:3) for 3 h. **(A, B)** The generation and fluorescence intensity fold of ROS in PMs was examined by flow cytometry assay and Rosup as a positive control. PMs were pretreated with NAC for 2 h, then infected with *N. caninum* (MOI 1:3) for 24 h. The production of IL-1β **(C)**, IL-6 **(D)**, IL-12p40 **(E)**, and IFN-γ **(F)** were detected by ELISA. One-way ANOVA assay with Tukey-Kramer *post hoc* test was used for analyzing the production of ROS and cytokines. Data are expressed as the mean ± SD from three independent experiments (**p<0.01, ***p<0.001, ns represents no significant differences).

ROS contributed to the production of proinflammatory cytokines (23) and mitophagy played an important role in ROS scavenging (16, 24). To examine whether *N. caninum*-induced mitophagy inhibited the production of proinflammatory cytokines through scavenging ROS in PMs, we detected the level of *N. caninum-*induced ROS in PMs treated with Mdivi1 and CCCP. Mdivi1 treatment enhanced the ROS generation in PMs infected by *N. caninum* (1.12-fold compared to DMSO-Nc group), while CCCP treatment suppressed it (78% compared to DMSO-Nc group) (**Figures 6A, B**). And the production of IL-1β, IL-6, IL-12p40, and IFN-γ were significantly reduced in *N. caninum*-infected PMs treated by NAC (**Figures 6C–F**). These data suggested that ROS, which could be controlled by *N. caninum*-induced mitophagy, down-regulated the production of IL-1β, IL-6, IL-12p40, and IFN-γ in PMs.

### 3.5 *N. caninum*-Induced Mitophagy Suppressed Nlrp3 Inflammasome and ERK Activations While Promoted the p38 Signal Pathway by Reducing ROS Generation in PMs

**Figure 7:**
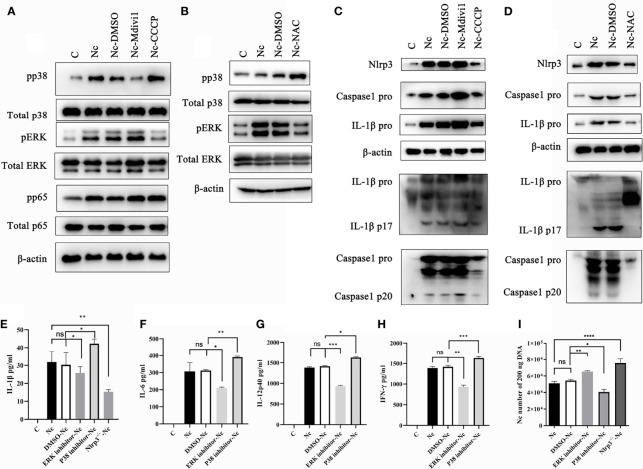
Mitophagy-mediated ROS generation controlled p38, ERK, and Nlrp3 inflammasome signals in PMs during *N. caninum* infection. WT PMs were pretreated with Mdivi1 (20 µM), CCCP (10 µM), and NAC (2 mM) for 2 h, and infected with *N. caninum* (MOI 1:3) for 30 min. **(A)** The phosphorylations of p38, ERK, NF-κB p65 were measured by Western blot in WT PMs pretreated with Mdivi1 (20 µM) and CCCP (10 µM). **(B)** The phosphorylation of p38 and ERK in PMs pretreated with NAC were detected by Western blot. WT PMs were pretreated with Mdivi1 (20 µM), CCCP (10 µM), and NAC (2 mM) for 2 h, and infected with *N. caninum* (MOI 1:3) for 24 h. **(C, D)** The expressions and cleavage of Caspase1, IL-1β, and Nlrp3 were detected by Western blot. WT PMs were pretreated with SB230580 and PD98059 for 2 h and infected with *N. caninum* (MOI 1:3) for 24 h. The production of IL-1β **(E)**, IL-6 **(F)**, IL-12p40 **(G)**, and IFN-γ **(H)** were detected by ELISA. The WT PMs pretreated with SB230580 and PD98059 and Nlrp3^-/-^ PMs were treated with *N. caninum* (MOI 1:3) for 24 h. **(E)** The production of IL-1β was measured by ELISA and **(I)** the number of parasites was evaluated by qPCR. One-way ANOVA assay with Tukey-Kramer *post hoc* test was used for analyzing the number of *N. caninum* and production of cytokines. Data are expressed as the mean ± SD from three independent experiments (*p<0.05, **p<0.01, ***p<0.001, ****p<0.0001, ns represents no significant differences).

It is noted that Nlrp3, NF-κB, p38, and ERK signal pathways participated in modulating the production of inflammatory cytokines (4, 31–33). Therefore, we examined whether Nlrp3 inflammasome, NF-κB, p38, and ERK signal pathways in *N. caninum-*infected PMs were modulated by the mitophagy-ROS axis. The data revealed the phosphorylation of ERK was reduced in *N. caninum*-infected PMs treated with CCCP and NAC, while that was up-regulated in Mdivi1-treatment group (**Figures 7A, B**). On the contrary, the phosphorylation of p38 was promoted by NAC or CCCP, but was inhibited in PMs treated by Mdivi1 during *N. caninum* infection (**Figures 7A, B**). However, there were no significant differences in the phosphorylation of NF-κB p65 in PMs treated by CCCP and Mdivi1 during *N. caninum* infection (**Figure 7A**). The Western blot results showed that NLRP3, pro-caspase1, pro-IL-1β, cleavage of caspase-1, and active IL-1β were significantly increased after Mdivi1 treatment while decreased after CCCP or NAC treatment in *N. caninum*-infected PMs for 24 h (**Figures 7C, D**). To further investigate the roles of the p38, ERK, and Nlrp3 inflammasome in cytokine production and parasite proliferation, p38 (SB203580) and ERK (PD98059) inhibitors and Nlrp3^-/-^ mice PMs were used. SB203580 treatment promoted *N. caninum*-induced production of IL-1β (42.29 ± 2.51 pg/ml), IL-6 (391.81 ± 9.71 pg/ml), IL-12p40 (1626.45 ± 30.74 pg/ml), and IFN-γ (1630.32 ± 65.91 pg/ml) in PMs compared to solvent-*N. caninum* group (30.31 ± 6.74 pg/ml, 311.89 ± 11.34 pg/ml, 1414.56 ± 30.33 pg/ml, 1387.21 ± 74.32 pg/ml). While PD98059 treatment inhibited *N. caninum*-induced production of IL-1β (25.71 ± 3.59 pg/ml), IL-6 (209.72 ± 10.15 pg/ml), IL-12p40 (931.17 ± 41.12 pg/ml), and IFN-γ (935.82 ± 71.74 pg/ml) in PMs (**Figures 7E–H**). The production of IL-1β (15.29 ± 2.12 pg/ml) was inhibited in Nlrp3^-/-^ PMs infected by *N. caninum* (**Figure 7E**). Correspondingly, the number of parasites was increased in PD98059-treated PMs (1.17-fold) and Nlrp3^-/-^ PMs (1.39-fold), while the number of parasites was decreased in SB203580-treated PMs (75%) (**Figure 7I**). Taken together, *N. caninum*-induced mitophagy-ROS axis could regulate the production of cytokines through p38, ERK, and Nlrp3 inflammasome pathways.

## 4 Discussion

Mitochondrial damage usually occurred during microbial infection or cellular stress, subsequently, the damaged mitochondria were eliminated by mitophagy to maintain mitochondrial stability (18, 24, 34, 35). Mitophagy exhibits decreased levels of Tim23, Hsp60, and mtDNA/nDNA ratios (20). Host cell mitophagy has been found during certain viral and bacterial infections (18–20, 34). However, host mitophagy occurrence has not been reported during parasite infection. In the present study, autophagosomes and co-localization of LC3 with mitochondria were observed in *N. caninum-*infected host cells, and mtDNA/nDNA ratios, mitochondrial marker proteins were decreased. In addition, the levels of *N. caninum*-induced mitophagy were increased with increased parasite numbers and extended infection time, which was similar with *Hepatitis c* virus non-structural protein 5A-induced mitophagy and *classical swine fever* virus-induced mitophagy (36, 37). *N. caninum* ESPs did not trigger mitophagy occurrence. However, the ESPs secreted by *N. caninum* under pressure in macrophages might not exactly overlap with the ESPs of the parasite without pressure, suggesting that it is not certain whether mitophagy can be induced by *N. caninum* through secreting protein in PMs. Taken together, these results indicated that host cell mitophagy occurred in vitro and in vivo during *N. caninum* infection. In our knowledge, this is the first report that host cell mitophagy was observed in parasites infection.

When mitophagy inducer (CCCP) was used to promote mouse mitophagy, *N. caninum* infected mice exhibited shortened survival time, decreased body weight, and increased parasite load, which indicated that *N. caninum* exacerbated the disease process through triggering mitophagy. Previous researches have shown that *L. monocytogenes*, *influenza A* virus, *Hepatitis B* virus, and *human immunodeficiency* virus could induce mitophagy to attenuate innate immune system for viral and bacterial persistence (18–20, 34). The inflammatory cytokines such as IL-1β, IL-6, IL-12p40, and IFN-γ played critical roles in anti-*N. caninum* infection (4–6). Promoting mice mitophagy inhibited the secretions of proinflammatory cytokines during *N. caninum* infection. Some protozoa have evolved various strategies to evade host immunity (7, 8). Inhibiting inflammatory cytokines production is a key mechanism for protozoan immune evasion. For example, *Toxoplasma gondii* could control the signaling pathways of host immunity such as STAT1, NF-κB signals, and caspase-1 cleavage to decrease the production of cytokines such as IFN-γ and IL-1β (9). The present data showed that *N. caninum*-triggered mitophagy could assist parasites to escape from host clearance, which is a novel mechanism of protozoan immune evasion.

Mitochondrial dysfunction could result in ROS release into the cytosol and mitophagy occurs to optimize clearance of abnormal mitochondria to decrease ROS accumulation in the cytosol (38). The production of ROS could be inhibited by mitophagy in *Influenza* viruses and *Escherichia* infection (18, 39–41). In agreement with previous studies, our study revealed that promoting mitophagy could reduce the production of ROS in *N. caninum* infection, while inhibiting mitophagy up-regulated ROS production. ROS was a critical regulator of the splenic response (phagocytes, T cells, and cytokines) to *T. cruzi* infection (42) and ROS up-regulated cytokine expressions in cardiomyocytes infected by *T. cruzi* (43). Uncoupling protein 2 negatively regulated mitochondrial ROS generation to regulate the production of cytokines in experimental visceral leishmaniasis (44). We found that NAC inhibited ROS generation to suppress the production of inflammatory cytokines during *N. caninum* infection.

It is noted that p38, ERK, and Nlrp3 inflammasome regulated proinflammatory cytokines production (4, 13, 22, 28, 33). Moreover, previous studies clarified that p38/ERK signals and Nlrp3 inflammasome were involved in *N. caninum* infection (4, 13, 22, 28, 33). ROS has been reported to activate NF-κB activity in cells (45). *Cryptococcus heimaeyensis* S20 exopolysaccharide-induced ROS could regulate p38 and ERK signals to trigger autophagic cell death in lung cancer cells (46). Koumine induced ROS generation to suppress hepatocellular carcinoma cell proliferation *via* NF-κB and ERK/p38 signals (47). *N. caninum-*induced ROS generation was involved in NETs formation and Nlrp3 inflammasome activation against *N. caninum* infection (30, 48). We found that *N. caninum*-induced mitophagy could regulate ROS-mediated ERK/p38 signals and Nlrp3 inflammasome. However, the phosphorylation level of NF-κB p65 was not profoundly altered in PMs through promoting or inhibiting *N. caninum*-induced mitophagy. The present data clarified the detailed mechanism of the mitophagy-ROS axis involved in *N. caninum* infection.

In conclusion, *N. caninum* promoted host mitophagy to attenuate the production of proinflammatory cytokines in a ROS-dependent manner through regulating the activations of p38, ERK, and Nlrp3 inflammasome signals, which revealed a novel immune evasion mechanism of *N. caninum*. The role of host mitophagy in other parasites need to be explored in the future.

## Data Availability Statement

The original contributions presented in the study are included in the article/**Supplementary Material**. Further inquiries can be directed to the corresponding author.

## Ethics Statement

The animal study was reviewed and approved by Animal Welfare and Research Ethics Committee at Jilin University.

## Author Contributions

Manuscript writing: XZ, XL, XCZ, and JL. Data analysis: XZ, YW, and XL. Experimental methodology: XZ, YW, PG, and XW. Experimental design: XZ, NZ, RW, and JL. Experimental guiding: XCZ, MC, and JL. Manuscript revisions: XZ, XL, and JL. All authors contributed to the article and approved the submitted version.

## Funding

This Research was funded by the National Basic Science Research Program (973 program) of China (No. 2015CB150300). The experiments conducted in this study comply with the current laws of China.

## Conflict of Interest

The authors declare that the research was conducted in the absence of any commercial or financial relationships that could be construed as a potential conflict of interest.

## Publisher’s Note

All claims expressed in this article are solely those of the authors and do not necessarily represent those of their affiliated organizations, or those of the publisher, the editors and the reviewers. Any product that may be evaluated in this article, or claim that may be made by its manufacturer, is not guaranteed or endorsed by the publisher.

## Supplementary Material

Supplementary Figure 1 Co-localization of LC3 with mitochondria were observed in *N. caninum-*stimulated PMs by IFA. PMs were stimulated with *N. caninum* at MOI 1:3 for 16 h, with CCCP (10 µM) treatment as the positive control, and the medium only as the negative control. **(A)** The number of PMs in which endogenous LC3 co-localized with Mito Tracker were evaluated per 50 cells. One-way ANOVA assay with Tukey-Kramer *post hoc* test was used for analyzing the numbers of PMs. **(B)** 293T cells were transfected with pEGFP-LC3 vector and pEGFP empty vector for 24 h by using Lipofectamine 2000 transfection reagent. Then the cells were stimulated with *N. caninum* at MOI 1:3 for 16 h. The number of 293T cells in which GFP-LC3 co-localized with Mito tracker were counted per 50 cells. Unpaired, two-tailed Student’s T-test was analyzed for the number of 293T cells. Data are expressed as the mean ± SD from three independent experiments (*p<0.05, **p<0.01, ***p<0.001, ns represents no significant differences).

## Abbreviations

ESPs, *N. caninum* excretory secretion production; ROS, Reactive Oxygen Species; qPCR, quantitative real-time PCR; ELISA, enzyme−linked immunosorbent assay; FBS, fetal bovine serum; MOI, multiplicities of infection; PBS, phosphate buffer saline; PBST, phosphate−buffered saline with tween−20; DAMPs, damage-associated molecular patterns; mtDNA, mitochondrial DNA; WT, wild type; DMSO, Dimethyl sulfoxide; TEM, transmission electron microscopy; nDNA, nucleic DNA; PMs, peritoneal macrophages.
