# Supplementary figures and images for: Neospora caninum Evades Immunity via Inducing Host Cell Mitophagy to Inhibit Production of Proinflammatory Cytokines in a ROS-Dependent Manner
Source: Front Immunol. 2022 Mar 9;13:827004. doi: 10.3389/fimmu.2022.827004 (PMC8959673; doi:10.3389/fimmu.2022.827004)

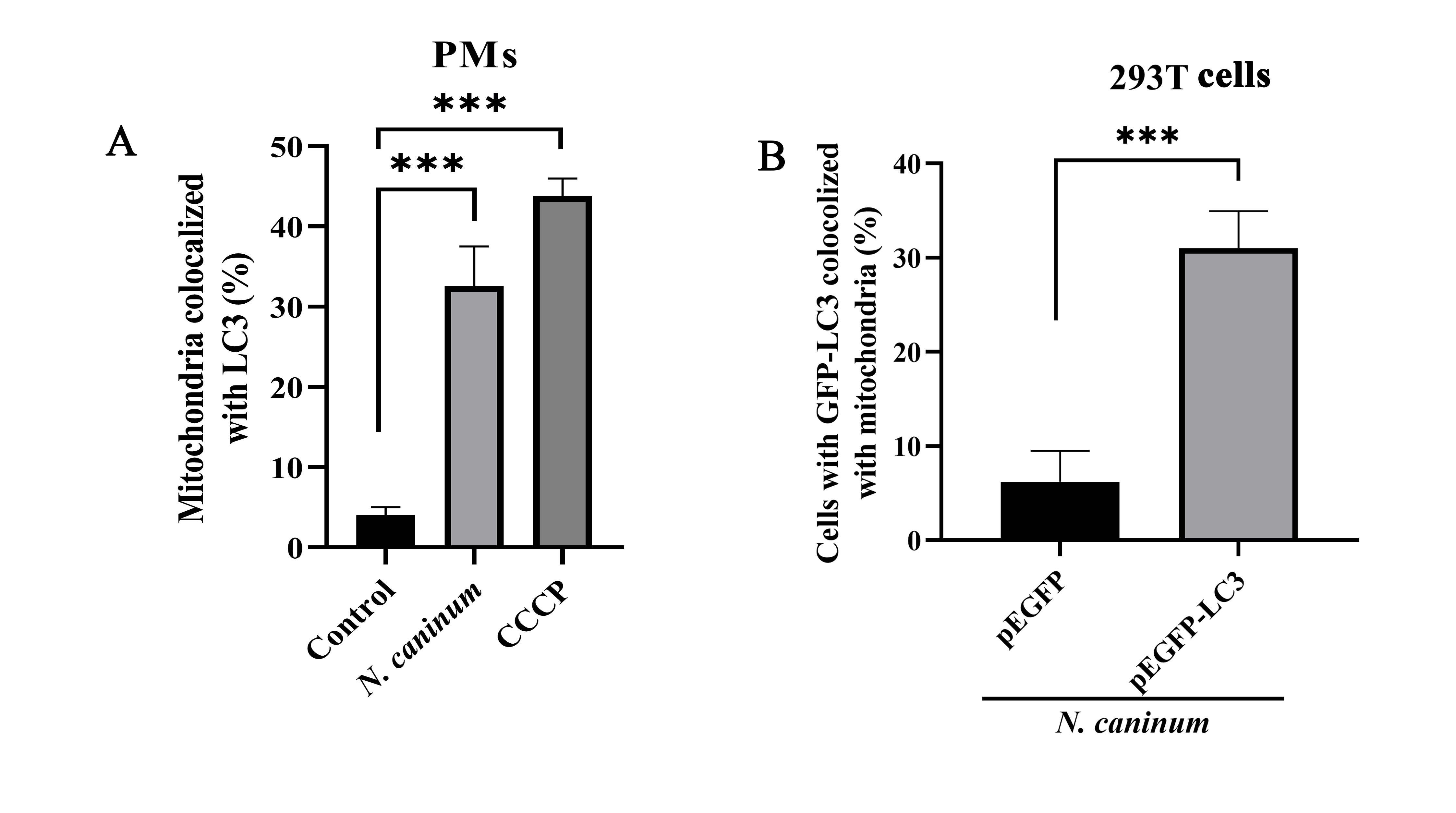

Supplement: Supplementary Figure 1 — Co-localization of LC3 with mitochondria were observed in N. caninum-stimulated PMs by IFA. PMs were stimulated with N. caninum at MOI 1:3 for 16 h, with CCCP (10 µM) treatment as the positive control, and the medium only as the negative control. (A) The number of PMs in which endogenous LC3 co-localized with Mito Tracker were evaluated per 50 cells. One-way ANOVA assay with Tukey-Kramer post hoc test was used for analyzing the numbers of PMs. (B) 293T cells were transfected with pEGFP-LC3 vector and pEGFP empty vector for 24 h by using Lipofectamine 2000 transfection reagent. Then the cells were stimulated with N. caninum at MOI 1:3 for 16 h. The number of 293T cells in which GFP-LC3 co-localized with Mito tracker were counted per 50 cells. Unpaired, two-tailed Student’s T-test was analyzed for the number of 293T cells. Data are expressed as the mean ± SD from three independent experiments (*p<0.05, **p<0.01, ***p<0.001, ns represents no significant differences). [file Image_1.tif]
